# Clinical Situations of Bacteriology and Prognosis in Patients with Urosepsis

**DOI:** 10.1155/2019/3080827

**Published:** 2019-02-06

**Authors:** Ying Jiang, Jun Li, Yingrui Zhang, Xinlan Hu, Xiaoguang Zhang, Xiuling Shang, Shurong Gong, Rongguo Yu

**Affiliations:** ^1^Department of Surgical Critical Care Medicine, Fujian Provincial Hospital, Fujian Provincial Clinical Teaching Hospital Affiliated to the Fujian Medical University, Fuzhou, Fujian, China; ^2^Microbiology Department of Fujian Provincial Hospital, Fujian Provincial Clinical Teaching Hospital Affiliated to the Fujian Medical University, Fuzhou, Fujian, China

## Abstract

**Background:**

Urosepsis and septic shock are a critical situation leading to a mortality rate up to 30% in patients with obstructive diseases of the urinary tract.

**Aim:**

To analyze the bacterial distribution and drug resistance of pathogenic bacteria in patients with urosepsis and to provide a basis for the rational application of antibacterial drugs in clinical practice.

**Methods:**

A retrospective analysis of 94 hospitalized patients with urosepsis for 6 years was performed. The strain composition, resistance characteristics, and the antibiogram of common bacteria from positive blood and midstream urine culture were analyzed.

**Results:**

A total of 87 strains were isolated, including 65 strains (74.71%) of Gram-negative bacilli, 14 strains (16.09%) of Gram-positive cocci, and 8 strains (9.20%) of fungi. The Gram-negative bacilli included 42 strains of* Escherichia coli* (*E. coli*) (64.62%), among which 34 strains (80.95%) were producing ESBLs, and 14 strains (21.84%) of* Klebsiella pneumoniae* (*K. pneumoniae*), among which nine strains (64.29%) were producing ESBLs. The most common pathogenic bacteria, ESBL+* E. coli* and* K. pneumoniae* strains, showed sensitivity towards imipenem, ertapenem, piperacillin/tazobactam, amikacin, and cefotetan, but were highly resistant to quinolones. The cure rate of urosepsis was 88.30%, and the susceptibility rate of septic shock was 45.47%.

**Significance:**

Gram-negative bacterial infections are the main cause of urosepsis. The mild patient group showed more* E. coli* (ESBL-) infections, and the number of ESBL producing* E. coli* isolated from the mild group showed higher drug resistance rates for aztreonam and levofloxacin compared with isolates from the severe group.

## 1. Introduction

Sepsis is a global public health problem, and it is also one of the most common critical infectious diseases, with a mortality rate as high as 20-42%. Approximately 215,000 patients in the United States die of septic shock every year, among which 9.1% were infected with an etiological source from the urogenital system [1]. Urosepsis is a life-threatening organ dysfunction resulting from systemic metabolic imbalance in response to the infection, which normally originates from the urogenital tract of the host [2].

Urinary system diseases, including urinary tract obstruction and associated iatrogenic surgical injury, may often predispose the patients to develop secondary infections of varying etiology [3]. Due to the complexity of urinary tract obstruction, secondary infections may occur in the presence of urethral stones or hydronephrosis that lead to the formation of bacterial biofilms. Second, many invasive surgical procedures such as local puncture of the urinary system can cause serious damage to the normal skin and mucous membrane barriers [4]. Most of the current surgical methods are based on intraluminal invasive procedures and, for example, percutaneous nephrolithotomy (PCNL) involving high pressure irrigation and exosmose of the irrigating fluid can lead to the destruction of the tissue structure [5]. Other invasive procedures such as transrectal prostate biopsy can lead to damage of the intestinal mucosal barrier, and the intestinal flora entering the blood can increase the risk of sepsis and subsequent septic shock. Once the urinary tract infection progresses into urinary septic shock, the mortality rate is greatly increased [6].

A number of studies have been conducted to explore the epidemiological characteristics of urinary tract infections and sepsis, but there is still a lack of relevant bacteriological characteristics and prognostic analysis of urosepsis in China [7]. Some global research reports suggested that the most common pathogenic bacteria isolated from nosocomial urosepsis caused by urinary tract infections were mostly* Escherichia coli*,* Enterococci*,* Pseudomonas aeruginosa*, and* Klebsiella spp.*, which are highly resistant to the routinely used antibiotics (among these pathogenic microorganisms, 8% were resistant to imipenem and 62% to beta-lactamase inhibitors) [8]. Although the European guidelines for urinary tract infections provided valuable guidance on the diagnosis and treatment of various urinary tract infections, there are still great differences in the pathogenic spectrum and susceptibility results of urosepsis around the world [9].

Clinically, in terms of the principles for antibacterial treatment in urosepsis patients with unknown etiology, stratified diagnosis of the risk factors for drug-resistant bacteria should be performed based on the epidemiological data of local monitoring and assessment of pathogens and its drug resistance and to initiate and perform timely empirical treatment [10]. This study retrospectively analyzed the distribution and drug resistance characteristics of common pathogenic bacteria isolated from blood and urine of patients with urosepsis in a hospital in China. The outcomes and prognosis of infected patients were analyzed. We hoped to predict the relationship between the clinical severity of urosepsis and the pathogenic bacteria and their drug resistance, in order to help the clinicians improve their understanding and clinical prognosis of patients with urosepsis. Moreover, our analysis may provide theoretical basis and relevant data for the rational use of antibiotics and guide the clinical treatment of urinary tract infections.

## 2. Patients and Methods

### 2.1. Patients

A retrospective study of data collected from the patients with urosepsis who were admitted to the Department of Urology and ICU, Fujian Provincial Hospital, from January 2012 to December 2017, was conducted. The study protocol was approved by the hospital ethics committee.

#### 2.1.1. Inclusion Criteria

Urinary tract infections were diagnosed according to the Health Industry Standards of the People's Republic of China WS/T 489-2016 “Laboratory Diagnosis of Clinical Microbiology of Urinary Tract Infections”: the number of pathogenic bacteria in culture of clean catch midstream urine or catheterized urine specimen was ≥10^5^ CFU/L. Urosepsis was diagnosed according to the 2017 European Guidelines for Urinary Tract Infection: life-threatening organ dysfunction caused by imbalanced response to the infection in the host induced by urinary tract infections. Urosepsis can be divided into three stages: systemic inflammatory response syndrome (SIRS), sepsis, and septic shock.

#### 2.1.2. Exclusion Criteria

(1) Patients with comorbid infection in other sites, such as pulmonary infection, catheter-related bloodstream infections (CRBSI, referred to bloodstream infection and bacteremia caused by the bacteria and fungi that colonized an intravascular indwelling catheter), abdominal infection or intracranial infections, etc.; or (2) hospitalized patients due to other infections.

### 2.2. Diagnostic Criteria and Grouping

According to the severity of the disease, the included patients were divided into two groups: mild and severe. Urosepsis patients with early manifestation of systemic inflammatory response syndrome (SIRS) were classified as the mild group and those who progressed to sepsis or septic shock was classified as the severe group.

Systemic inflammatory response syndrome (SIRS) is a systemic nonspecific inflammatory response caused by severe damage due to infectious or noninfectious factors such as infection, trauma, burns, surgery, ischemia-reperfusion, etc., eventually leading to a cluster of clinical symptoms of uncontrolled inflammatory response in the body. Systemic reactions caused by severe infections include changes in body temperature, respiration, heart rate, and white blood cell count. The characteristics include body temperature >38°C or <36°C, heart rate >90 beats/min, respiratory rate >20 beats/min or partial pressure of arterial carbon dioxide <32 mmHg, peripheral white blood cell count >12,000/mm^3^ or <4000/mm^3^.

Sepsis was defined as a life-threatening organ dysfunction caused by imbalanced response to the infection in the host, which can be indicated by the sequential organ failure assessment (SOFA) score in the clinical setting (SOFA ≥2 points) [11]. The quick sequential organ failure assessment (qSOFA) score is composed of three items: changes in conscious state, systolic blood pressure ≤100 mmHg, and respiratory frequency ≥22 times/min. Patients who meet two or more of these criteria items, i.e., qSOFA score ≥2) were categorized as suspected sepsis cases.

The clinical diagnostic criteria for septic shock recommended in the guidelines are persistence of hypotension after full capacity resuscitation in patients with sepsis; administration of vasoconstrictive drugs to maintain the mean arterial pressure (MAP) ≥65 mmHg; and serum lactate levels >2 mmol/L.

### 2.3. Sample Collection

Urine and blood samples from all subjects were collected, separated, and cultured in accordance with the Chinese Health Industry Standards. During the study period, blood culture, identification of bacteria, and drug sensitivity analysis were performed with the use of the BacT/Alert 3D fully automatic blood culture system (BioMerieux, France), which supports aerobe and anaerobe culture flasks, and of a VITEK 2 MS mass spectrometer, which is a fully automatic bacteria identification system. The system also allows for drug sensitivity identification, which was performed using Columbia blood agar, Maconkey agar, and MH agar (Beiruite Biotechnology (Zhengzhou) LLC, Zhengzhou, China) and drug sensitive paper (Oxoid Co., UK). Inoculation, isolation, and culture were conducted for all specimens in accordance with the National Clinical Laboratory Procedures (3rd edition). The judgment criteria were based on the Clinical and Laboratory Standards Institute (CLSI). Quality-control bacterial strains included* Pseudomonas aeruginosa* ATCC27853,* Escherichia coli *ATCC25922,* Klebsiella pneumoniae *ATCC700603,* Staphylococcus aureus *ATCC25923, and* Staphylococcus aureus *ATCC25913.

### 2.4. Survey Methods

A retrospective survey approach was used. The inpatient medical records were reviewed and data were collected for patients who met the criteria including age, gender, length of hospital stay, primary diseases in urinary system, underlying diseases, comorbidities, pathogenic examinations, bacterial culture, and hospital mortality.

### 2.5. Statistical Analysis

The data presented as means ± standard deviation, medians and range, and frequencies and percentage, as appropriate. Continuous data were analyzed using the *t*-test or Mann-Whitney U test, as appropriate. Categorical data were analyzed using the chi-square test. Two-sided P values <0.05 were considered as statistically significant. Data were analyzed using the Statistical Package for Social Sciences (SPSS) version 16.0 (IBM, Armonk, NY, USA)

## 3. Results

**Figure 1:**
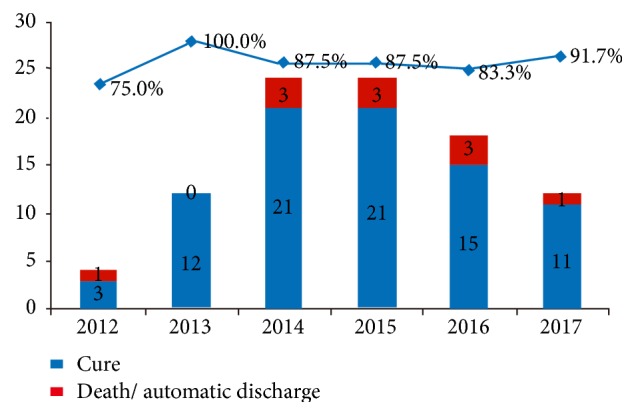
Cure rate versus mortality rate among the patients, per year from 2012 to 2017. Highest cure rate (100%) was noted during 2013. And the lowest cure rate (75%) was in 2012.

A total of 94 eligible patients who met the inclusion criteria were included; 47 males and 47 females, aged 17-88 years with an average of 59.83 years. Among them, 83 cases were cured, 10 cases were initiatively discharged, and one case died (Figure 1).

The 94 patients were classified into the mild group (38 cases) and severe group (56 cases). Among the mild cases, the male/female ratio was 24/14 with an average age of 58.09 years. Among severe cases, the male/female ratio was 23/33, and the average age was 62.39 years (Table 1). Of the 94 patients with urosepsis secondary to infection of urinary system, 30 cases were hospitalized in the ICU (seven mild cases and 23 severe cases). The occurrence rate of urinary sepsis shock was 45.47% (Table 1). Among the patients with urosepsis, 52 cases (57.45%) were secondary to the urinary tract obstruction with stones or hydronephrosis; 38 cases (40.43%) were due to urinary surgery.

### 3.1. Etiologic Distribution

The blood and urine samples were collected and a total of 87 different bacterial strains belonging to 22 species were identified. Among them, there were 65 strains (74.71%) of eight species of Gram-negative bacteria; 14 strains (14.09%) of nine species of Gram-positive bacteria, and eight strains (9.2%) of five species of fungi. The most common Gram-negative bacteria isolated from patients with urosepsis were* E. coli* (ESBLs+/-),* K. pneumoniae* (ELBSs-/+),* Enterobacter cloacae*,* Stenotrophomonas maltophilia*,* Proteus mirabilis*,* Pseudomonas aeruginosa*,* Acinetobacter baumannii*, and* Acinetobacter junii*. The most common Gram-positive bacteria isolated from patients with urosepsis were* Enterococcus faecium*,* Enterococcus faecalis*,* Staphylococcus epidermidis*,* Staphylococcus capitis*, human* Staphylococcus* subgroup,* Staphylococcus saprophyticus*,* Aerococcus viridians*, and* Staphylococcus warneri*. The fungi included* Candida albicans*,* Candida parapsilosis*,* Candida tropicalis*,* Candida glabrata*, and* Trichosporon asahii* (Tables 2–4). The severe group showed more ESBL nonproducing* Escherichia coli* (*E. coli* ESBL-) isolates compared with the mild group (P<0.05).

### 3.2. Gram-Negative Bacteria

The detection rates of extended-spectrum *β*-lactamases (ESBLs) in* E. coli* and* K. pneumoniae* strains were 80.95% and 64.29%, respectively. The resistance rates of the main pathogenic bacteria in this group, ESBLs-producing* E. coli* strain (E. coli+), were all higher than 80% to most antibiotics such as ampicillin (penicillin), cefazolin (first generation cephalosporin), ceftriaxone (third-generation cephalosporin), ciprofloxacin and levofloxacin (quinolones). The drug resistance rates of this strain were 50% to aztreonam (monocyclic amides) and cefepime (fourth-generation cephalosporins). The drug resistance rates of ESBLs-producing* K. pneumonia* strain (*K. pneumonia* +) were also higher than 90% to antibiotics such as ampicillin-sulbactam, cefazolin, ceftriaxone, gentamicin, and trimethoprim/sulfamethoxazole. The drug resistance rates of the two strains were higher than 50% to gentamicin and tobramycin (aminoglycosides). Their sensitivity to carbapenems, such as imipenem and ertapenem were 100%, and they were relatively sensitive to enzyme inhibitor compound drugs such as piperacillin/tazobactam.

### 3.3. Gram-Positive Bacteria

The Gram-positive bacteria were mainly* Enterococcus faecium* and* Staphylococcus*. The drug resistance patterns of both* Enterococcus faecium* and* Staphylococcus* in the mild and severe groups were compared, but did not show any significant difference among them (Supplement Table 1). The drug resistance rates of* Enterococcus faecium* (accounting for 66.66% of the Gram-positive strains) were greater than 50% to most of the antibiotics. It was 100% resistant to antibiotics such as ampicillin, penicillin G, ciprofloxacin, levofloxacin, and moxifloxacin. One case of drug resistance to vancomycin was sensitive to linezolid and tigecycline (Table 4).

Drug resistance of* Escherichia coli* strains isolated from the mild and severe groups of patients were analyzed. The number of ESBL producing (ESBL+)* E. coli*, showing drug resistance to aztreonam (100% versus 68.75%) and Levofloxacin (100% versus 75%) isolated from the mild group was higher than in the severe groups (P<0.05). Regarding the ESBL+/-* Escherichia coli* isolated in the two groups, no other significant difference in antibiotic resistance to any other antibiotics was observed (Table 5). This may be due to the small sample size in this retrospective study.

## 4. Discussion

Urinary tract infections (UTIs) are the second most common infectious diseases after respiratory infections. Urosepsis is a life-threatening organ dysfunction caused by imbalanced response to the infections in the host due to urinary tract originated infections. It can be manifested as three stages: early SIRS, sepsis, and septic shock [9]. Patients with urosepsis can progress from almost innocuous state to severe sepsis in a very short period of time. Therefore, patients with urosepsis, especially those with complex urinary tract infections, should be diagnosed, intervened, and treated at an early stage. SIRS is commonly recognized as the first event in a cascade leading to multiple organ failure, with a significant increase in mortality [12, 13]. Treatment strategy for urosepsis should include appropriate life-sustaining and prompt antimicrobial therapy, supplementary measures, and optimal management of urinary system disorders [14]. Urosepsis is more common in men than in women, with a higher rate of detection in elderly patients, and suffering with Gram-negative bacteria as the main pathogen. The local factors include urinary tract obstruction with urethral stones comorbid with hydronephrosis, high pressure washing with percutaneous nephroscopy, and transrectal biopsy [15].

According to the monitoring results in our hospital, the pathogenic bacteria were still dominated by Gram-negative bacteria. For the strains isolated from this retrospective study,* E. coli* was the main pathogenic bacteria (accounting for 61.67% of all pathogenic bacteria) among Gram-negative bacteria, which was close to the results from Nicolie et al. (42-69.3%) [16].

This study suggested that the detection rates of extended-spectrum *β*-lactamases (ESBLs) in* E. coli *and* K. pneumoniae* strains were 80.95% and 64.29%, respectively, which were higher than in previous reports. Indeed, the 2015 CHITE test data suggested that the detection rates of ESBLs in* E. coli* and* K. pneumoniae* were 51.5% and 27.4%, respectively [17]. The production of extended-spectrum enzymes ESBLs is one of the major resistance mechanisms of* E. coli* and* K. pneumoniae*. ESBLs are a class of plasmid-mediated *β*-lactamases that are able of hydrolyzing penicillins, oxyiminocephalosporins (including third- and fourth-generation cephalosporins), and monocyclic amides aztreonam, and can be inhibited by *β*-lactamase inhibitors [18]. Early studies on the resistance mechanism of ESBLs indicated that it is formed by the point mutation of 1-7 amino acids in the TEM-1 or SHV-1 molecular structure. In recent years, the genotype of ESBLs has greatly changed. The CTX-M type had replaced the TEM and SHV as the main genotypes of ESBLs, accounting for more than 70% of all ESBLs genotypes [19]. ESBL producing strains often simultaneously carry the drug resistance genes for aminoglycosides, tetracyclines, and quinolones, which allow the spread of drug resistance genes among bacteria by binding, transformation, and transduction, resulting in occurrence of severe intrahospital cross-infection and spread of resistant bacteria outside the hospital.

*E. coli* and* K. pneumoniae* are the most common ESBLs-producing bacteria in the* Enterobacteriaceae* family, followed by* Proteus* bacteria [20]. The main pathogenic bacteria in this group, ESBLs-producing* E. coli* strains, had drug resistance rates of >80% to most antibiotics such as ampicillin (penicillins), cefazolin (first generation cephalosporins), ceftriaxone (third-generation cephalosporins), ciprofloxacin, and levofloxacin (quinolones). The drug resistance rates were 75% to aztreonam (monocyclic amides) and cefepime (fourth-generation cephalosporins). The resistance rates of ESBLs-producing* K. pneumoniae* strain were more than 80% to antibiotics such as ampicillin-sulbactam, cefazolin, ceftriaxone, gentamicin, and trimethoprim/sulfamethoxazole (Supplement Table 2). The resistance rates of these two strains were greater than 50% to gentamycin and tobramycin (aminoglycoside).

Some previously published studies [21–23] showed that the main risk factors for ESBLs-producing bacterial infections included repeated use of antimicrobial drugs, indwelling catheters (including central vein or arterial catheters, percutaneous gastric or jejunostomy fistulas), the presence of urethral stones or obstructions (biliary/urinary tract), previous ESBLs-producing bacterial infections, repeated hospitalizations (including care centers), previous admission in ICUs, elderly patients, underlying diseases (diabetes, immune dysfunction), and ventilator-assisted breathing. Meanwhile, studies have shown that the increase of mortality in patients with bloodstream infection due to ESBLs-producing enteric bacterial infection was not associated with the production of ESBLs, but was due to the inappropriate empiric treatment in patients with community-acquired bloodstream infections caused by ESBLs strains, suggesting the importance of appropriate empirical treatment for patients with sepsis [20].

In this study, the drug resistance rate of urosepsis pathogenic spectrum to quinolones was as high as 90%. Therefore, for patients with high risk factors for urosepsis (such as urinary tract stones combined with obstruction, operation of endoluminal lithotripsy, etc.), the quinolone group of drugs such as levofloxacin should be carefully selected as the first choice for the empirical administration of drugs in the early phase of treatment. Further clinical data are required for clarifying whether a combination drug therapy for ESBLs-producing strains can be recommended if* in vitro* susceptibility tests show the sensitivity to relevant bacterial isolate. At the same time, the drug resistance rates of ESBLs-producing strains to third- and fourth-generation cephalosporins are as high as 75% [24]. In order to reduce the multiplication and spread of ESBLs-producing strains and *β*-lactam antibiotic resistance, the use of third-generation cephalosporins should be restricted to perioperative prophylactics for patients with low risk for ESBLs-producing strains, and they should be avoided in empirical anti-infection regimens. Carbapenem antibacterial drugs (imipenem and ertapenem) had shown high antibacterial activity against ESBLs-producing* E. coli* and* K. pneumoniae *strains, and are currently the most effective and reliable antibacterial drugs in the treatment of infections caused by ESBLs-producing* Enterobacteriaceae* group [25]. However, with the increasing use of this class of drugs, even though it showed a high stabilizing effect on *β*-lactamase and strong antibacterial activity, it had been reported that the corresponding drug-resistant strains had emerged [25]. Meanwhile, it was easy to cause dysbacteriosis and secondary fungal infection due to the indiscriminate use of broad spectrum antibacterial drugs [26].

In this study, the drug resistance rate of ESBLs-producing strains to *β*-lactam inhibitors was less than 10%. Therefore, in order to avoid the abusive use of carbapenems in clinical practice, the carbapenems (imipenem, ertapenem, and meropenem) are only recommended in patients with high risk of extended-spectrum *β*-lactamase (ESBL) strains. For some patients, the *β*-lactam inhibitors may also be selected. In this study, when the extent of infection with different pathogenic bacteria was compared between the two groups, the mild group showed more ESBL nonproducing* E. coli* compared with the severe groups. A significantly high drug resistance against aztreonam (100% versus 68.75%) and levofloxacin (100% versus 75%) were shown by ESBL producing (ESBL+)* E. coli* strains isolated from the mild group compared with the strains from the severe group, which is consistent with a previous study [27].

The major limitation of the study is the small sample size, which could preclude an exact interpretation of specific patterns of drug resistance in the mild and severe group of patients. This could be solved by conducting a multicenter study involving a large number of patients.

## 5. Conclusion

Gram-negative bacterial infections are the most common cause of urosepsis in our study. When the mild and severe groups were compared for extent of infections with different bacterial pathogens, the mild group showed more* E. coli* (ESBL-) infections compared with the severe groups. Regarding the drug resistance pattern, the number of ESBL producing (ESBL+)* E. coli* isolated from the mild group showed higher drug resistance rates for aztreonam and levofloxacin, compared with isolates from the severe group. Treatment strategy for urosepsis should include appropriate life-sustaining and prompt antimicrobial therapy, supplementary measures, and optimal management of urinary system disorders. Quinolones should be selected as the first choice for the empirical administration of drugs in the early phase of treatment. Carbapenem antibacterial drugs should be the first-line treatment against ESBLs-producing* E. coli* and* K. pneumoniae *strains. In order to reduce the multiplication and spread of ESBLs-producing strains and *β*-lactam antibiotic resistance, the use of third-generation cephalosporins should be restricted to perioperative prophylactics for patients with low risk for ESBLs-producing strains, and they should be avoided in empirical anti-infection regimens.

## Figures and Tables

**Table 1:** Clinical data of the patients.

	Mild (n=38)	Severe (n=56)	P
Age (years) (median, interval )	58 (17-76)	62 (27-88)	0.1660
Gender (male/female ratio)	24/14	23/33	0.0356
Background diseases			
Diabetes	7	11	0.8825
Hypertension	14	22	0.8110
Chronic kidney disease	6	2	0.0372
Chronic heart disease	5	2	0.0823
Chronic lung disease	2	1	0.0347
Cerebrovascular disease (coma)	1	0	0.0222
Autoimmune disease	0	4	0.0922
Gestation	1	0	0.0222
Urinary tract stone obstruction with hydronephrosis	13	34	0.3997
High pressure washing with percutaneous nephroscopy	1	3	0.5206
Transrectal biopsy	10	3	0.0039
Hospital stay (days)	21 (7-68)	36 (3-100)	0.2038
Number of hospitalization in ICU (cases)	7	23	0.0208
ICU stay (days)	10.14	19.56	0.8051
Time from admission to episode of sepsis	6.53 (0-20)	4.87 (0-52)	0.3693
Indwelling catheter (cases)	15	52	<0.0001
Time from episode of sepsis to indwelling catheter	12±31.57	11±20.00	0.3041

#, one case of catheter-related infection for 365 days.

**Table 2:** Distribution of pathogens isolated from mild and severe cases.

	Total		Mild (n=42)		Severe (n=45)		P
	Bacterial strains (strain)	Proportion (%)	Bacterial strains (strain)	Proportion (%)	Bacterial strains (strain)	Proportion (%)	
Gram-negative bacteria	65	74.71	31	73.81	34	75.56	0.9912
* E. coli* (+)	34	39.08	18	42.86	16	35.56	0.4855
* K. pneumoniae* (+)	9	10.34	4	9.52	5	11.11	0.8081
* E. coli* (-)	8	9.2	1	2.38	7	15.56	0.0336
* K. pneumoniae* (-)	5	5.75	3	7.14	2	4.44	0.5889
* Proteus mirabilis*	2	2.3	0	0	2	4.44	NA
* Stenotrophomonas maltophilia*	2	2.3	2	4.76	0	0	NA
* Pseudomonas aeruginosa*	2	2.3	2	4.76	0	0	NA
* Acinetobacter baumannii*	1	1.15	0	0	1	2.22	NA
* Acinetobacter junii*	1	1.15	0	0	1	2.22	NA
* Enterobacter cloacae*	1	1.15	1	2.38	0	0	NA
Gram-positive bacteria	14	16.09	7	16.67	7	15.54	0.8879
* Enterococcus faecium*	5	5.75	3	7.14	2	4.44	0.5889
* Enterococcus faecalis*	1	1.15	1	2.38	0	0	NA
* Streptococcus alactolyticus*	1	1.15	0	0	1	2.22	NA
* Aerococcus viridans*	1	1.15	0	0	1	2.22	NA
Human *Staphylococcus* subgroup	2	2.3	0	0	1	2.22	NA
* Staphylococcus epidermidis*	1	1.15	1	2.38	1	2.22	NA
* Staphylococcus capitis*	1	1.15	0	0	1	2.22	NA
* Staphylococcus saprophyticus*	1	1.15	1	2.38	0	0	NA
* Staphylococcus warneri*	1	1.15	1	2.38	0	0	NA
Fungi	8	9.2	4	9.52	4	8.89	0.9183
* Candida albicans*	4	4.6	3	7.14	1	2.22	0.6244
* Candida glabrata*	1	1.15	1	2.38	0	0	NA
* Candida parapsilosis*	1	1.15	0	0	1	2.22	NA
* Trichosporon asahii*	1	1.15	0	0	1	2.22	NA
* Candida tropicalis*	1	1.15	0	0	1	2.22	NA

(+): ESBL producing; (-): ESBL nonproducing bacterial isolate.

**Table 3:** Drug resistance of Gram-negative bacteria.

	*E. coli* (+)		*K. pneumoniae* (+)		*E. coli* (-)		*K. pneumoniae* (-)	
	n	Drug resistance rate%	n	Drug resistance rate%	n	Drug resistance rate%	n	Drug resistance rate%
Ampicillin	28	80	4	50	2	22.22	1	20
Ampicillin-sulbactam	25	71.43	2	25	8	88.89	1	20
Aztreonam	23	65.71	1	12.5	5	55.56	0	0
Cefazolin	32	91.43	1	12.5	9	100	0	0
Ceftriaxone	32	91.43	1	12.5	9	100	0	0
Ceftazidime	14	40	1	12.5	2	22.22	0	0
Cefotetan	0	0	0	0	1	11.11	0	0
Cefepime	18	51.43	0	0	1	11.11	0	0
Piperacillin/tazobactam sodium	2	5.71	0	0	1	11.11	0	0
Imipenem	0	0	0	0	0	0	0	0
Ertapenem	0	0	0	0	0	0	0	0
Ciprofloxacin	33	94.29	4	50	5	55.56	3	60
Levofloxacin	32	91.43	4	50	3	33.33	1	20
Amikacin	6	17.14	0	0	1	11.11	0	0
Gentamicin	19	54.29	3	37.5	9	100	3	60
Tobramycin	14	40	2	25	5	55.56	1	20
Trimethoprim/sulfamethoxazole	21	60	5	62.5	9	100	3	60

**Table 4:** Drug resistance of two Gram-positive bacteria of *Enterococcus* spp.

	*Enterococcus faecium*		*Enterococcus faecalis*	
	n	Drug resistance rate%	n	Drug resistance rate%
Ampicillin	5	100.0%	-	-
Penicillin G	5	100.0%	6	100.0%
Benzylpenicillin	-	-	5	83.3%
Vancomycin	1	20.0%	0	0.0%
Teicoplanin	1	20.0%	0	0.0%
Linezolid	0	0.0%	0	0.0%
Ciprofloxacin	5	100.0%	2	33.3%
Levofloxacin	5	100.0%	1	16.7%
Moxifloxacin	5	100.0%	0	0.0%
Clindamycin/chloramphenicol	2	40.0%	5	83.3%
Erythromycin	3	60.0%	6	100.0%
Gentamicin	-	-	2	33.3%
High-level gentamicin	4	80.0%	0	0.0%
High-level streptomycin	1	20.0%	0	0.0%
Tetracycline	3	60.0%	1	16.7%
Tigecycline	0	0.0%	-	-

**Table 5:** Drug resistance of *E. coli* (ESBL +/-) in mild and severe patients.

	ESBLs + (n=34)					ESBLs - (n=8)				
	Mild		Severe		P	Mild		Severe		P
	n=18	%	n=16	%		n=1	%	n=7	%	
Ampicillin	14	77.78	15	93.75	0.1893	0	0	4	57.14	NA
Ampicillin-sulbactam	12	66.67	13	81.25	0.3360	0	0	2	28.57	NA
Aztreonam	18	100	11	68.75	0.0102	0	0	1	14.29	NA
Cefazolin	16	88.89	16	100	0.1693	0	0	1	14.29	NA
Ceftriaxone	16	88.89	16	100	0.1693	0	0	1	14.29	NA
Ceftazidime	6	33.33	8	50	0.3243	0	0	1	14.29	NA
Cefotetan	0	0	0	0	NA	0	0	0	0	NA
Cefepime	9	50	9	56.25	0.7155	0	0	0	0	NA
Piperacillin/tazobactam sodium	2	11.11	0	0	NA	0	0	0	0	NA
Imipenem	0	0	0	0	NA	0	0	0	0	NA
Ertapenem	0	0	0	0	NA	0	0	0	0	NA
Ciprofloxacin	18	100	15	93.75	0.2817	1	100	3	42.86	NA
Levofloxacin	18	100	12	75	0.0239	1	100	3	42.86	NA
Amikacin	3	16.67	3	18.75	0.8736	0	0	0	0	NA
Gentamicin	12	66.67	7	43.75	0.1792	0	0	3	42.86	NA
Tobramycin	8	44.44	6	37.5	0.6813	0	0	2	28.57	NA
Trimethoprim/sulfamethoxazole	11	61.11	10	62.5	NA	1	100	4	57.14	NA

## Data Availability

The data used to support the findings of this study are available from the corresponding author upon request.

## Supplementary Materials

Supplementary Materials Supplement Table 1: the drug resistance of Gram-positive bacteria in mild and severe groups. Supplement Table 2: the drug resistance rates of K. pneumoniae in the mild and severe groups.
