# Supplementary material for: Clinical Situations of Bacteriology and Prognosis in Patients with Urosepsis
Source: Biomed Res Int. 2019 Feb 6;2019:3080827. doi: 10.1155/2019/3080827 (PMC6381567; doi:10.1155/2019/3080827)
Supplement: Supplementary Materials — Supplement Table 1: the drug resistance of Gram-positive bacteria in mild and severe groups. Supplement Table 2: the drug resistance rates of K. pneumoniae in the mild and severe groups. [file 3080827.f1.zip › 3080827.f1/3080827.f1.docx]

Supplement . Table 1. The drug resistance of Gram-positive bacteria in mild and severe groups

|  | *Enterococcus faecium* | | | | *Staphylococcus* | | | |
| --- | --- | --- | --- | --- | --- | --- | --- | --- |
|  | Mild | | Severe | | Mild | | Severe | |
|  | Drug resistant strain (n=3) | Drug resistance rate (%) | Drug resistant strain (n=2) | Drug resistance rate (%) | Drug resistant strain (n=3) | Drug resistance rate (%) | Drug resistant strain (n=3) | Drug resistance rate (%) |
| Ampicillin | 3 | 100 | 2 | 100 | - | - | - | - |
| Penicillin G | 3 | 100 | 2 | 100 | 3 | 100 | 3 | 100 |
| Benzylpencilline | - | - | - |  | 2 | 66.7 | 3 | 100 |
| Vancomycin | 0 | 0 | 1 | 50 | 0 | 0 | 0 | 0 |
| Tecolanin | 0 | 0 | 1 | 50 | 0 | 0 | 0 | 0 |
| Linezolid | 0 | 0 | 0 | 0 | 0 | 0 | 0 | 0 |
| Ciprofloxacin | 3 | 100 | 2 | 100 | 0 | 0 | 2 | 66.7 |
| Levofloxacin | 3 | 100 | 2 | 100 | 0 | 0 | 1 | 33.3 |
| Moxifloxacin | 3 | 100 | 2 | 100 | 0 | 0 | 0 | 0 |
| Clindamycin/chloram | 1 | 33.3 | 1 | 50 | 2 | 66.7 | 3 | 100 |
| Erythromycin | 2 | 66.7 | 1 | 50 | 3 | 100 | 3 | 100 |
| Gentamicin | - | - | - | - | 1 | 33.3 | 1 | 33.3 |
| High-level gentamicin | 3 | 100 | 1 | 0 | 0 | 0 | 0 | 0 |
| High-level streptomycin | 1 | 50 | 0 | 0 | 0 | 0 | 0 | 0 |
| Tetracycline | 2 | 66.7 | 1 | 50 | 0 | 0 | 1 | 33.3 |
| Tigecycline | 0 | 0 | 0 | 0 | - | - | - | - |
