# Supplementary material for: Clinical Situations of Bacteriology and Prognosis in Patients with Urosepsis
Source: Biomed Res Int. 2019 Feb 6;2019:3080827. doi: 10.1155/2019/3080827 (PMC6381567; doi:10.1155/2019/3080827)
Supplement: Supplementary Materials — Supplement Table 1: the drug resistance of Gram-positive bacteria in mild and severe groups. Supplement Table 2: the drug resistance rates of K. pneumoniae in the mild and severe groups. [file 3080827.f1.zip › 3080827.f1/3080827.f2.docx]

S. Table 2. The drug resistance rates of *K. pneumoniae* in the mild and severe groups

| Antibiotics | ESBLs + (n=9) | | ESBLs – (n=5) | |
| --- | --- | --- | --- | --- |
|  | Mild | Severe | Mild | Severe |
| Ampicillin | 50.00 | 0.00 | 33.33 | 0.00 |
| Ampicillin-sulbactam | 100.00 | 80.00 | 33.33 | 0.00 |
| Aztreonam | 75.00 | 40.00 | 0.00 | 0.00 |
| Cefazolin | 100.00 | 100.00 | 0.00 | 0.00 |
| Ceftriaxone | 100.00 | 100.00 | 0.00 | 0.00 |
| Ceftazidime | 25.00 | 20.00 | 0.00 | 0.00 |
| Cefotetan | 25.00 | 0.00 | 0.00 | 0.00 |
| Cefepime | 0.00 | 20.00 | 0.00 | 0.00 |
| Piperacillin/tazobactam sodium | 25.00 | 0.00 | 0.00 | 0.00 |
| Imipenem | 0.00 | 0.00 | 0.00 | 0.00 |
| Ertapenem | 0.00 | 0.00 | 0.00 | 0.00 |
| Ciprofloxacin | 75.00 | 40.00 | 100.00 | 0.00 |
| Levofloxacin | 75.00 | 0.00 | 33.33 | 0.00 |
| Amikacin | 25.00 | 0.00 | 0.00 | 0.00 |
| Gentamicin | 100.00 | 100.00 | 100.00 | 0.00 |
| Tobramycin | 75.00 | 40.00 | 33.33 | 0.00 |
| Trimethoprim/sulfamethoxazole | 100.00 | 100.00 | 100.00 | 0.00 |
